# The Impact of COVID-19 on the Quality of Life and Happiness of Care Home Residents in Croatia: A Cross-Sectional Study

**DOI:** 10.3390/bs12110463

**Published:** 2022-11-20

**Authors:** Michael Olumekor, Andrea Stojić, Tatjana Kehler, Francesco Polo

**Affiliations:** 1 Graduate School of Economics and Management, Ural Federal University, 620014 Yekaterinburg, Russia; 2 Faculty of Medicine, University of Rijeka, 51000 Rijeka, Croatia; 3 Cultural Centre Humanitas in Conegliano, 31015 Treviso, Italy

**Keywords:** COVID-19, elderly, care homes, nursing homes, happiness, quality of life, well-being

## Abstract

Care/nursing homes globally have been severely affected by the COVID-19 pandemic and have disproportionately experienced a high rate of mortality which led to the introduction of strict isolation policies. However, while there are studies on the mortality, epidemiology, staffing challenges, and mismanagement in long-term care homes as a result of COVID-19, there appears to be a paucity of information regarding the Quality of Life (QoL), happiness, and associated well-being of the elderly residents of these homes. Therefore, we examined if COVID-19 affected the happiness level, QoL, and financial condition of long-term care home residents in Croatia. To achieve this, a survey of 308 participants in eight long term care homes was conducted. Descriptive analysis was performed to describe the mean of all responses and the Bayesian Integrated Nested Laplace Approximation (INLA) was used to provide a detailed quantitative analysis of the results. We found that the QoL and happiness of residents remained relatively stable during the COVID-19 pandemic. However, the income level, financial outlook, marital status, and vaccination positivity influenced the QoL and happiness of care home residents to a considerable degree. We recommend that policy makers pay attention to these underlying factors.

## 1. Introduction

Since the latter months of 2019, the Severe Acute Respiratory Syndrome Coronavirus-2 (SARS-CoV-2), popularly termed coronavirus disease 19 or COVID-19 has brought a devastating impact to our world [1]. Since it was declared a pandemic by the World health Organization, the John Hopkins University’s Coronavirus Resource Centre has reported more than 6.4 million global COVID-19 deaths as of 17 August 2022, and about 592 million cases [2]. These, alongside the recent troubling mutations of the virus, which include the Delta and Omicron variants, have often forced governments to impose/re-impose stringent restrictions to control the outbreak. There have been extensive scientific studies on COVID-19, including its immunopathology [3,4,5,6,7], pathophysiology [1,8,9,10,11,12,13], neuropathology [14,15,16,17,18], epidemiology [19,20,21,22], and symptomatology [23,24,25,26], among others. 

While COVID-19 can be fatal to people across all age groups, there is an almost unanimous conclusion from scientists that it disproportionately affects the elderly. For example, in one of the most consequential studies on this, a research on 72,314 people in mainland China showed that the severity and fatality of the virus increased as people became older [27] and other empirical studies have buttressed this [27,28,29,30,31,32]. Moreover, as evidence mounted on the increased susceptibility of elderly people to COVID-19, many countries introduced measures to isolate and protect them [21,22,23,24,25,33,34]. Research is currently inconclusive on whether isolating the elderly might have led to increased mental distress among them or not. For example, in an extensive survey of mental health in the United States during the pandemic, Czeisler et al. [35] found that elderly people were far less likely to turn to substance abuse as a result of the pandemic. They also found that elderly people were less likely to suffer from trauma-or stress-related disorder and depressive disorder, among others. These findings were similar to surveys conducted in Spain and other countries showing no pandemic-related mental health problems among the elderly [36,37,38]. 

However, other studies have reached different conclusions. Other studies have called for a more nuanced interpretation of the literature on the topic, calling for more studies looking into the long-term impact on the well-being of the elderly [39]. There are also strong arguments connecting the isolation policies of governments to challenges in elderly mental and physical health [40,41]. Furthermore, Quality of Life (QoL) can be challenging to define, measure, and incorporate into scientific discourse due to its diverse health, philosophical, social, economic, and political definitions [42]. When it is health-related, the core components of QoL are multidimensional, comprising functional, emotional/psychological, physical, and social/occupational [42] measuring QoL can be challenging [43]. Moreover, elderly adults are not a monolith, and a disproportionate number of studies have shown that socio-economic factors, demographics, and other characteristics influences their QoL and overall mental well-being during the pandemic [35,44,45,46,47,48,49]. 

In addition, the COVID-19 pandemic did not just disproportionately affect elderly people in general, it had an even more devastating impact on elderly residents of care home facilities. An extensive study of 22 countries by the International Long-Term care Policy Network found that on average, 41% of all COVID-19-related deaths, 325,000 deaths, were residents of care homes [50]. In fact, there is an almost unanimous agreement by scientists on the enormously difficult impact of the pandemic on care homes [51,52,53,54,55]. For example, as of October 2021, 30,130 care home residents in the United Kingdom had died from the COVID-19 pandemic. In Spain it was 30,170, in Germany it was 23,750, in France it was 30,000, in Belgium it was 12,899, and in the United States it was 137,126 [56]. However, while there have been extensive studies on mortality [50,52,55,56], epidemiology [53], staffing challenges [57], and mismanagement [51] in care home facilities, there are very few studies specifically looking into the QoL and happiness/subjective well-being of care home residents during the pandemic. Furthermore, the importance of care homes to the social fabric of many developed societies, and the vulnerability of their residents, also make this research a timely contribution to the debate. 

Therefore, the aim of this research was to examine if COVID-19 affected the happiness level, QoL, and financial condition of long-term care home residents in Croatia. To our knowledge, there have been very few prior studies specific to the mental well-being of care home residents. As such, this paper hopes to make the following practical contributions. First, it provides valuable insights which are helpful for doctors, mental health professionals, and other caregivers, helping to improve their method of assessment and approach to working with care home residents in future pandemics. We also hope to provide insights for future isolation, restriction, and lockdown policies by thoroughly examining the impact of these policies on the mental well-being of care home residents. Furthermore, for academic research, this paper contributes to the growing debate on the impact of the pandemic on care homes. However, it differs from all the others by focussing on the analysis of the QoL and happiness level of care home residents. We hope this encourages future research on the topic. 

## 2. Materials and Methods

### 2.1. Research Process

Our research process includes scoping relevant literature, streamlining research questions, developing/grouping survey questions, ethical considerations, the survey approach, hypotheses formulation, and model development/analysis. The goal of our study was to investigate if/how COVID-19 might have impacted the happiness level, QoL, and financial condition of long-term care home residents in Croatia including eight (n = 8) care homes with a total of 308 residents.

The survey questionnaire was conceptualised following brainstorming sessions by researchers, which comprised social science and medical experts. In addition, research gaps were established following the scoping of relevant literature, before we proceeded to streamline the survey questions. The survey questions were adapted from the highly cited Oxford Happiness Questionnaire (OHQ) [58] and the newer COVID-19 specific COVID-19–Impact on Quality of Life scale (COV19–QoL) [59]. The unique nature of our study meant it was better to take a hybrid approach to our survey questions, adopting questions from both the OHQ developed for elderly people, and the COV19–QoL—developed for COVID-19, to suit the unique characteristics of our research subjects. In total, there were 30 survey questions (n = 30) structured using a 5-point Likert scale system ranking from strongly disagree to strongly agree, and organized into the five main categories of demographics, Quality of life, financial condition/outlook, vaccination and institutional trust, and happiness. We provide a succinct elaboration of the categories below:

Category 1 contained questions on demographics: (1) Your age. (2) Marital status. (3) Ethnicity. (4) Income level.

Category 2 contained questions on COVID-19 Quality of life: (5) Due to the spread of the COVID-19 I think my quality of life is lower than before. (6) Due to the spread of the COVID-19 I think my mental health has deteriorated. (7) Due to the spread of the COVID-19 I think my physical health may deteriorate. (8) Due to the spread of the COVID-19 I feel more tense than before. (9) Due to the spread of the COVID-19 I feel more depressed than before. (10) Due to the spread of the COVID-19 I feel that my personal safety is at risk. (11) Due to the spread of the COVID-19 I miss contact with my family and friends.

Category 3 contained questions on financial outlook: (12) I have experienced financial problems because of COVID-19. (13) I am worried about losing my pension and income because of COVID-19. (14) I know someone who has experienced financial problems because of COVID-19. (15) I am optimistic about my future financial condition.

Category 4 contained questions on vaccination which we also took to mean institutional trust: (16) I will take the COVID-19 vaccine if a healthcare professional recommends it. (17) After receiving the COVID-19 vaccine, I will feel safer. (18) After vaccination, I will hang out with friends and family without fear of COVID-19.

Category 5 contained questions on happiness: (19) I feel pleased with the way I am. (20) I am very interested in other people. (21) I feel that life is very rewarding. (22) I have very warm feelings towards almost everyone. (23) Life is good. (24) I think that the world is a good place. (25) I laugh a lot. (26) I am well-satisfied about everything in my life. (27) I am very happy. (28) My life is better compared with 1 year ago. (29) I would like more people to enjoy life with. (30) I feel optimistic about the future.

### 2.2. Ethical Considerations

Our research followed the Helsinki Declaration, as revised in 2013, and the recommendations of the International Committee of Medical Journal Editors (ICMJE). All participants were made aware of the nature of our research and they provided written informed consent to us. Our research received institutional ethical approval from the ethical review committee of the faculty of Health Studies, University of Rijeka, classification number: 600-05/22-01/37. 

### 2.3. Survey Approach, Hypotheses Formulation, and Preparation

Our research is based upon a survey of care home residents in Croatia. We utilised a mixed sampling approach beginning with a probabilistic clustered sampling in the first stage, before proceeding to a convenience sampling method in the second stage, due to the unprecedented challenges in care homes at the time. 

There were two groups of hypotheses. In the first group, two hypotheses were formulated based on the length and strictness of care home isolation policies:

**Hypothesis** **1:** Care home residents experienced less happiness because of COVID-19.

**Hypothesis** **2:** Care home residents experienced a reduction in their quality of life because of COVID-19.

In the second group, two hypotheses were formulated to account for the impact of socio-demographic and economic factors:

**Hypothesis** **3:** Characteristics such as income level, age, and marital status do not positively influence the QoL of care home residents.

**Hypothesis** **4:** Characteristics such as age, income level, marital status, financial outlook and vaccination positivity do not positively influence the happiness of care home residents.

After completing our survey preparation, we proceeded to recruit respondents for the research. To achieve this, we obtained the official directory and addresses of care homes in Croatia from the website of the Croatian Ministry of Labour, Pension System, Family, and Social Policy [60]. This directory contained copious details of care homes broken down into the various regions of Croatia. It included the names of the homes, the people responsible for managing each home, and the contact information, including emails and phone numbers, of every care home, among others. We then contacted the care homes asking them to assist us in carrying out our survey on their residents. Because we were not sure of the operational status of the phone numbers and email addresses on the government list we also directly contacted members of staff of several care home providers through publicly available information on their websites, and by referral from other care homes.

### 2.4. Study Setting and Participants

To select our study participants, we chose to include older adults, which we defined as people above 60 years of age, living in nursing homes or an institutional setting in Croatia during the COVID-19 pandemic. We excluded people less than 60 years of age, and people with health challenges such as physical or sensory disabilities and mental impairments such as dementia. A more detailed explanation of our exclusion and inclusion criteria is provided as a supplemental file. A total of 14 care homes were contacted. Of this number, we were only successful in recruiting 308 participants from 8 care homes across Croatia. They include the following: 2 care home in Istra county (43 participants), 3 care homes in Primorsko-Goranska county (181 participants), and 3 care homes in Grad Zagreb (84 participants). The names of the care homes can be provided on request. All residents of these homes who met our inclusion criteria and provided written consent were included in the research. Due to the nature of participants, our study made use of a paper-based survey by printing out our survey questions and sending them by post to the specific care home facility, and by delivering them by hand to a member of staff. Moreover, we also received the survey results the same way. 

We received a total of 308 responses (n = 308) and excluded a few responses which contained incomplete data and multiple answers to questions. As a result, 295 ideal responses (n = 295) were used for our analysis. 

### 2.5. Model Development, Coding and Analysis

The data were processed using Microsoft Excel (ver. 2019), Stata (ver. 2016), and R (>ver. 4) software. Excel was used to recode the collected numeric form, which was then transferred to Stata were the value labels were attached, before they were recoded into a more concise format for analysis. Thereafter, the dataset was moved to R where the Bayesian Integrated Nested Laplace Approximation (INLA) Logistic Regression was implemented.

The model was developed to provide a detailed analysis of our survey results. Our model was a Bayesian binary logistic regression fitted with the Integrated Nested Laplace Approximation (INLA) to estimate its posterior marginals. Logistic regression is useful for examining the relationship between binary outcomes and predictors. The method of maximum likelihood [61] is usually used for estimating the coefficients of the model from a frequentist inference perspective. A Bayesian inference approach however has become an increasingly preferred alternative. It produces highly accurate results and is computationally less complicated than other competing models [62]. Moreover, several prominent literatures have provided detailed explanations of the method, approach, benefits, accuracy, scope, and importance of fitting INLA to Bayesian logistic regression [61,62,63,64,65,66]. Furthermore, in the last decade there have been well-established studies using a similar model to analyse regional variations in excess cancer mortality [67], animal models in evolutionary biology [68], age-stratified cancer incidence and mortality [69], spatial analysis of disease clusters [70,71], analysis of child maltreatment [72], and the occurrence patterns of fish species [47], among others.

A Bayesian inference depends on prior information defined as prior probabilities, and the likelihood of the distribution of the outcome variable for estimating the coefficients of the model defined by posterior marginals. This means

(1) $$p\left(\theta ,y\right)=\frac{p\left(y,\theta \right)*\pi \left(\theta \right)}{p\left(y\right)}$$

where $p\left(\theta ,y\right)$ is the posterior density, $\pi \left(\theta \right)$ is the prior density, $p\left(y,\theta \right)$ is the probability distribution of the outcome variable, and $p\left(y\right)$ is a normalizing constant.

(1) can be simply expressed as Posterior=Likelihood×Prior.

From (1), $p\left(y\right)=\int_{\theta }=p\left(y,\theta \right)\pi \left(\theta \right)d\theta$ is called the marginal probability of the data under the model.

For binary responses such as the one we in this study, the experience of positive effect is denoted by (1) and the experience of negative effect is denoted by (0). The distribution of a binary response, say Y, which is Bernoulli-distributed with a success probability $P\left(Y=1\right)=\pi$ is given as:

$$f\left(y\right)=exp\left\{ylog\left(\frac{\pi }{1-\pi }\right)+log\left(1-\pi \right)\right\}$$

Using the logit function, the logistic regression can thus be written as:

$$\left\{\begin{array}{c} Y_{i}\sim Bernoulli\left(\pi_{i}\right)\\ logit\left(\pi_{i}\right)=\beta_{0}+\beta_{1}x_{i1}+\cdots +\beta_{p}x_{ip} \end{array}\right.$$

where $\beta_{i}$ represents the constant and coefficients of the predictors.

$x_{ip}$ = the predictors.

The coefficients are estimated as marginal probabilities using the Integrated Nested Laplace Approximation.

Laplace approximation is used to approximate integral $I_{n}=\int_{x}\exp\left(nf\left(x\right)\right)dx$ as n→∞.

By Taylor’s theorem

(2) $$I_{n}\approx exp\left(nf\left(x_{0}\right)\right)\sqrt{\frac{2\pi }{-nf^{\prime \prime }\left(x_{0}\right)}}$$

where $f^{\prime \prime }\left(x_{0}\right)$ denoted the second-order derivatives at $x_{0}$. $I_{n}$ is the Gaussian integral. If $nf\left(x\right)$ is interpreted as the sum of log-likelihoods and x as the unknown parameter, the Laplace approximation is exact as n→∞, if the central theorem holds (Rue et al., 2017).

When (2) is numerically applied, we have $\tilde{p}\left(\theta_{j},y\right)=\int \tilde{p}\left(\theta |y\right)d\theta_{-j}$, where $\theta_{-j}\;$ denotes the vector of θ with its *j*th element excluded, and $\tilde{p}(\theta^{\left(k\right)}|y)$ are the density values computed.

The posterior density computed at the end will take the form:

(3) $$\pi \left(x,\theta |y\right)\propto exp\left(-\frac{1}{2}x^{T}Q\left(\theta \right)x+\underset{i}{\sum }log\left(\pi \left(y_{i}|\eta_{i},\theta \right)\right)+log\pi \left(\theta \right)\right)$$

where x is a latent Gaussian field.

Assumptions for INLA are:

1. Each data point depends on only one of the elements in the latent Gaussian field x, the linear predictor so that the likelihood can be written as:
  $$y|x,\theta \sim \prod \pi (y_{i}|\eta_{i},\theta )$$
2. The size of the hyperparameter vector θ is small (say < 15).
3. The latent field x, can be large but it is endowed with some conditional independence properties so that the precision matrix $Q\left(\theta \right)$ is sparse.
4. The linear predictor depends linearly on the unknown smooth function of covariates.
5. The inferential interest lies in the univariate posterior marginals $\pi \left(x|y\right)$ and $\pi (\theta_{j}|y)$ rather than in the joint posterior π(x,θ|y) (Rue et al., 2009).

## 3. Results

### Sample Characteristics

**Figure 1:**
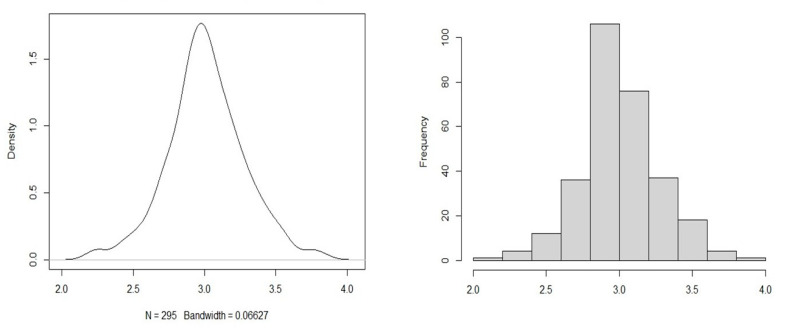
Spread of responses.

Figure 1 shows the density of responses to the survey questions. Of the 295 respondents, 14.5% were between the ages of 65 and 69, 17.6% between 70 and 74, 28.4% between 75 and 79, 21.3% between 80 and 84, 11.8% between 85 and 89, and 6.1% between 90 and 95. Additionally, 28.4% respondents were married, 15.5% were single, 27.7% were divorced, 26.4% had a deceased spouse, and 1.6% preferred not to reveal their marital statuses. For income level, 39.3% people reported a monthly income less than EUR 400, 37.6% between EUR 401 and EUR 800, 18.6% between EUR 801 and EUR 1000, 4% above EUR 1000, and one person preferred not to reveal their income. A total of 98.6% of the 295 respondents were of Croatian ethnicity. The other 1.4% chose ‘other European’ as their ethnicity. 

The mean of responses to QoL questions is shown in Table 1. The final row and column show the aggregate/overall *mean* of QoL responses. The results show an average overall mean (3.1). This implies that there were minor changes to the QoL of elderly residents of care homes due to COVID-19. Table 2 shows the calculated mean of responses to questions on happiness. The final row and column show the aggregate/overall mean for all happiness responses. The results from Table 2 show that the overall level of happiness, shown in the overall mean, appears average (3.1). This implies that there were not very significant changes to the level of happiness of care home residents due to COVID-19.

Furthermore, the explanatory variables are comprised of the following clusters: demographic variables, variables measuring the financial outlook of the respondents, and variables measuring a vaccination outlook (Table 3). These include the following:

Table 3 provides a detailed classification of the coding methodology. Two models were fitted for Quality of Life as an outcome variable while three variables were fitted for happiness as an outcome variable. All the fitted models were the Bayesian Logistic Regression estimated using INLA. 

Two outcome variables were considered in this study: Quality of Life (QoL) due to COVID-19, and happiness of care home residents. The two variables were measured multinomially on a Likert scale of strongly disagree (1) to strongly agree (5). The outcome variables were regrouped into binary forms to allow for the fitting of the Bayesian Integrated Nested Laplace Approximation Logistic Regression. In binary form, the categories are as follows:

1. Quality of Life due to COVID-19: Improved Quality of Life was coded as 1. Reduced Quality of Life was coded as 0.
2. Happiness of care home residents: Feel Happy was coded as 1. Feel Unhappy was coded as 0.

In achieving the above, the average of the scores for the items under each outcome variable was taken. An average greater than 3 was recoded as 1, an average equal to 3 was recoded as unsure, an average of less than 3 was recoded as 0. Data points with an average equal to 3 were dropped from the data set because it meant they were not up to 10% of the overall sample size. 

Table 4 shows the results of our QoL regression analysis of care home residents. The two models for QoL are Model I, which used the demographics data as its only explanatory variables, and Model II which used both demographics and financial outcomes as explanatory variables. Furthermore, for the results of our Bayesian INLA model, the likelihood of the effects of the explanatory variables on the outcome variables is represented by the Adjusted Odd Ratio (aOR) defined within a 95% confidence interval (C.I). (Table 4 and Table 5).

For QoL outcome, Model I shows that the income of care home residents earning between EUR 801–EUR 1000 significantly influenced the QoL of care home residents among other explanatory variables (aOR = 2.96, 95% CI = 1.27, 7.07). This outcome is significant as it suggests that respondents earning between EUR 801- EUR 1000 are two times more likely to have a good QoL, compared with people earning less than EUR 400 (Refer to Table 4). Model II reveals that financial outcome influenced the significance of the demographics in Model I, specifically, marital status and income level. For marital status, divorced care home residents are less likely to experience the effects of COVID-19, compared with single participants. Moreover, for income level, the odds of care home residents who earn between EUR 801–EUR 1000 are three times higher.

Additionally, for the outcome on happiness, Model I has no significant factor, but Model II shows that income level and financial outlook are significant. For income level in Model II, respondents who earn between EUR 401– EUR 800 are two times more likely to be happy compared with those who earn less than EUR 401 (aOR = 2.03, 95% CI:1.05, 3.99). Moreover, for financial outlook (refer to Table 5), those who experienced financial problems because of COVID-19 are two times more likely to be happy compared with those who experienced no financial problems. Furthermore, those who know someone who experienced financial problems because of COVID-19 and those who are not sure about this are three times and two times more likely to feel happy respectively, compared with those who do not. 

Model III revealed that residents 70–74 years of age have lower odds of feeling happy, compared with those aged 65–69. Income level was also significant in Model III because respondents earning between EUR 401–EUR 800 are three times more likely to feel happy compared with those who earning less than EUR 400. (Refer to Table 5) Furthermore, financial outlook was found to be insignificant under this model, whereas vaccination outlook was found to be significant. Participants who indicated that they would take the COVID-19 vaccine if a healthcare professional recommends it, and those who would feel safer after receiving the vaccine against COVID-19, are both three times more likely to feel happy. Similarly, those who would hang out with friends and family without fear of COVID-19 after receiving the vaccine are four times more likely to feel happy compared with those who would not.

Table 5 shows the regression results for happiness. Similar to the fitted models for QoL (Table 4), our analysis for happiness (Table 5) consists of Model I which also uses demographics as its only explanatory variables, and Model II which uses both demographics and financial outcomes as explanatory variables. However, it also has Model III (Table 5) which uses the trio of demographics, financial outcome, and vaccination outlook as its explanatory variables. Furthermore, these models were varied to separately examine how demographics affect the outcome variables (Model I) and to examine how the financial outlook modifies the effect of the demographics (Model II) and examine how financial outlook and vaccine collectively affect the effects of the demographics on the outcome variables (Model III) (Table 5). 

## 4. Discussion and Conclusions

This research surveyed how COVID-19 affected the happiness level, QoL, and financial condition of long-term care home residents in. Our research was conducted in eight care homes in Croatia, and we used the Bayesian INLA to provide a thorough analysis of the results. Our research did not find any significant impact to the QoL and happiness level of care/nursing home residents due to the COVID-19 pandemic. This supports the findings of many prior studies on the overall mental health of elderly people during the pandemic which showed that the mental health of older people was better than those of other age groups during the COVID-19 pandemic. For example, a study of the adult population in the United States revealed that older adults, people above 65 years of age, suffered less mental health challenges than younger adults [35]. A separate study involving older adults in The Netherlands revealed that while loneliness increased for elderly people, their mental health remained relatively stable during the pandemic [65]. Furthermore, a study in Spain by García-Fernández et al. [37] comparing symptoms of anxiety, depression, and acute stress between people above 60 years of age and people below, showed that people above 60 years of age had lower levels of mental distress than people under 60 years of age. This was buttressed by another study in Spain [36] which looked into PTSD, depression, and anxiety among Spanish adults. They found that people in the older age group were less likely to suffer from these symptoms. According to Vahia et al. [39], older adults tend to have “better emotional regulation and well-being than younger adults”.

However, our results are in contrast with several other studies in other countries, including in Sweden, Japan, Albania, Brazil, and others [44,45,46,47,48,49]. The research in Sweden was conducted between April and May 2020, and looked into the mental health of elderly Swedish adults [45]. They found that up to half of all the respondents reported a decrease in their mental health. In addition, studies on Japanese elderly adults examining the impact of decreased physical activity on subjective well-being, and the health-related quality of life of community-dwelling older people found that it worsened during the pandemic [47,49]. We speculate that socio-cultural factors and the level of isolation policies could have played a role in the disparity between the mental well-being of elderly adults across the world. Nevertheless, we strongly recommend future studies on this. 

Furthermore, all the aforementioned results on QoL in Model I and II and for happiness (Table 4 and Table 5) appear to support previously reported COVID-19 studies which found that factors such as age, socio-economic, demographic, and others can influence the mental well-being of elderly people [35,39,40,44,45,46,47,48,49,65]. For example, the findings of Czeisler et al. [35] showed that unemployed workers and minority groups were more likely to suffer mental health challenges during the pandemic, while Gustavsson and Beckman [45] found that the marital status of elderly people influenced their mental health during the pandemic. Additionally, having close friends and family can influence elderly mental health [40], gender played a role in the mental health of people during the pandemic [46], and the age group/bracket elderly adults belonged to influenced their quality of life [47]. 

To the best of our knowledge, this research is among the few to specifically examine the QoL and happiness of elderly residents of care homes. Our results provide a clear assessment of the impact of COVID-19 and isolation policies on the mental well-being of our research participants. It provides useful insights to healthcare practitioners in care homes, and for policy-makers in preparing for not just the current COVID-19 pandemic, but for future outbreaks as well. Methodologically, our assessment is also among the first specifically designed to measure QoL and happiness in care homes during a pandemic. We strongly recommend future work on this due to the very specific needs/characteristics of care home residents. However, our study is not without limitations. It is a cross-sectional study, in part due to the extraordinary level of restriction in Croatian care homes at the time of our research. Therefore, while our results might not be definitive or generalisable to other parts of the world, it provides an excellent baseline for understanding the impact of COVID-19 on care homes. We therefore recommend future research on the topic.

## Figures and Tables

**Table 1:** Mean responses to the Quality of Life of care home residents.

Quality of Life of Care Home Residents	Mean
Due to the spread of the COVID-19 I think my quality of life is lower than before.	2.6
Due to the spread of the COVID-19 I think my happiness has deteriorated.	3.1
Due to the spread of the COVID-19 I think my physical health may deteriorate.	3.3
Due to the spread of the COVID-19 I feel more tense than before.	3.0
Due to the spread of the COVID-19 I feel more depressed than before.	3.1
Due to the spread of the COVID-19 I feel that my personal safety is at risk.	3.1
Due to the spread of the COVID-19 I miss contact with my family and friends.	3.3
**Overall Mean**	**3.1**

**Table 2:** Mean of responses to the happiness of care home residents.

Happiness	Mean
I feel pleased with the way I am.	3.1
I am very interested in other people.	2.9
I feel that life is very rewarding.	3.2
I have very warm feelings towards almost everyone.	3.1
Life is good.	3.0
I think that the world is a good place.	3.0
I laugh a lot.	3.1
I am well-satisfied about everything in my life.	2.9
I am very happy.	3.2
My life is better compared with 1 year ago.	2.9
I would like more people to enjoy life with.	3.3
I feel optimistic about the future.	3.1
**Overall Mean**	**3.1**

**Table 3:** Coding methodology.

Characteristic	Categories	Code
**Demographics**		
**Age**	65–69	1
	70–74	2
	75–79	3
	80–84	4
	85–89	5
	90–95	6
**Marital status**	Single	1
	Married	2
	Divorced	3
	Deceased spouse	4
**Ethnicity**	Other European	0
	Croatian	1
**Income level**	Less than EUR 400	1
	EUR 401–EUR 800	2
	EUR 801–EUR 1000	3
	Above EUR 1000	4
**Financial Outlook**	**Coded as:**	
I have experienced financial problems because of COVID-19.		
I am worried about losing my pension and income because of COVID-19.	No	1
I know someone who has experienced financial problems because of COVID-19.	Not sure	2
I am optimistic about my future financial condition.	Yes	3
**Vaccine**	Coded as:	
I will take the COVID-19 vaccine if a healthcare professional recommends it.	No	1
After receiving the COVID-19 vaccine, I will feel safer.	Not sure	2
After vaccination, I will hang out with friends and family without fear of COVID-19.	Yes	3

**Table 4:** Regression results for the Quality of Life of care home residents.

	Quality of Life	
Characteristics	Model I	Model II
Demographics	aOR (95% CI)	aOR (95% CI)
**Age**		
65–69	Ref.	
70–74	1.32 (0.49, 3.54)	1.84 (0.61, 5.63)
75–79	0.49 (0.20, 1.23)	0.82 (0.28, 2.39)
80–84	0.55 (0.21, 1.42)	1.15 (0.39, 3.42)
85–89	0.56 (0.19, 1.60)	0.85 (0.25, 2.83)
90–95	0.99 (0.26, 3.82)	1.89 (0.39, 9.46)
**Marital status**		
Single	Ref.	
Married	0.55 (0.23, 1.30)	0.52 (0.19, 1.41)
Divorced	0.35 (0.14, 0.87)	0.31 (0.11, 0.88)
Deceased spouse	0.60 (0.24, 1.44)	0.53 (0.19, 1.48)
**Ethnicity**		
Other European	Ref.	
Croatian	2.19 (0.20, 36.42)	3.06 (0.05, 281.46)
**Income level**		
Less than EUR 400	Ref.	
EUR 401–EUR 800	1.77 (0.95, 3.33)	1.67 (0.82, 3.44)
EUR 801––EUR 1000	2.96 (1.27, 7.07)	3.01 (1.11, 8.41)
Above EUR 1000	3.67 (0.92, 17.32)	3.50 (0.66, 21.50)
**Finance Situation**		
**I have experienced financial problems because of COVID-19**		
No	Ref.	
Not Sure	-	2.08 (0.87, 5.10)
Yes	-	0.50 (0.22, 1.11)
**I am worried about losing my pension and income because of COVID-19**		
No	Ref.	
Not Sure	-	0.83 (0.35, 1.93)
Yes	-	0.30 (0.12, 0.75)
**I know someone who has experienced financial problems because of COVID-19**		
No	Ref.	
Not Sure	-	0.22 (0.10, 0.46)
Yes	-	0.17 (0.07, 0.39)
**I am optimistic about my future financial condition**		
No	Ref.	
Not Sure	-	0.48 (0.22, 1.06)
Yes	-	0.28 (0.12, 0.64)

Note: the parts highlighted in bold show the main sub-sections of our analysis.

**Table 5:** Regression results for the happiness of care home residents.

		Happiness	
Characteristics	Model I	Model II	Model III
Demographics	aOR (95% CI)	aOR (95% CI)	aOR (95% CI)
**Age**			
65–69	Ref.		
70–74	0.53 (0.20, 1.41)	0.43 (0.15, 1.19)	0.28 (0.09, 0.86)
75–79	0.91 (0.37, 2.23)	0.52 (0.19, 1.37)	0.37 (0.12, 1.10)
80–84	1.36 (0.52, 3.53)	0.91 (0.33, 2.54)	0.66 (0.21, 1.98)
85–89	1.10 (0.39, 3.12)	0.75 (0.24, 2.26)	0.76 (0.22, 2.59)
90–95	0.81 (0.21, 3.04)	0.50 (0.12, 2.14)	0.34 (0.07, 1.68)
**Marital status**			
Single	Ref.		
Married	0.69 (0.29, 1.63)	0.65 (0.25, 1.63)	0.92 (0.33, 2.53)
Divorced	1.08 (0.43, 2.68)	1.07 (0.40, 2.87)	1.16 (0.39, 3.39)
Deceased spouse	0.65 (0.27, 1.56)	0.59 (0.22, 1.55)	0.56 (0.19, 1.60)
**Ethnicity**			
Other European	Ref.		
Croatian	-	-	-
**Income level**			
Less than EUR 400			
EUR 401––EUR 800	1.52 (0.82, 2.83)	2.03 (1.05, 3.99)	3.21 (1.55, 6.89)
EIR 801––EUR 1000	0.51 (0.22, 1.17)	0.58 (0.23, 1.45)	1.12 (0.40, 3.10)
Above EUR 1000	0.42 (0.09, 1.69)	0.68 (0.12, 3.29)	1.05 (0.12, 7.68)
**Finance Situation**			
**I have experienced financial problems because of COVID-19**			
No	Ref.		
Not Sure	-	1.49 (0.68, 3.32)	1.93 (0.78, 4.85)
Yes	-	2.74 (1.31, 5.81)	2.22 (0.96, 5.16)
**I am worried about losing my pension and income because of COVID-19**			
No	Ref.		
Not Sure	-	0.69 (0.31, 1.51)	0.96 (0.40, 2.32)
Yes	-	0.67 (0.28, 1.58)	0.75 (0.29, 1.90)
**I know someone who has experienced financial problems because of COVID-19**			
No	Ref.		
Not Sure	-	2.40 (1.20, 4.88)	1.97 (0.91, 4.30)
Yes	-	3.41 (1.54, 7.72)	2.01 (0.81, 5.04)
**I am optimistic about my future financial condition**			
No	Ref.		
Not Sure	-	0.73 (0.36, 1.49)	0.66 (0.29, 1.46)
Yes	-	1.75 (0.84, 3.72)	2.19 (0.97, 5.09)
Vaccination			
**I will take the COVID-19 vaccine if a healthcare professional recommends it**			
No	Ref.	-	
Not Sure	-	-	1.91 (0.68, 5.53)
Yes	-		3.28 (1.14, 9.64)
**After receiving the COVID-19 vaccine, I will feel safer**			
No	Ref.	-	
Not Sure	-	-	1.51 (0.61, 3.72)
Yes	-		3.05 (1.24, 7.59)
**After vaccination, I will hang out with friends and family without fear of COVID-19**			
No	Ref.		
Not Sure	-	-	1.39 (0.57, 3.35)
Yes	-	-	4.72 (1.92, 11.83)

## Data Availability

Our research materials and data are accessible at the Open Science Framework: https://osf.io/k6ntx/?view_only=9334afd207c94969a659ec6de77c94db accessed on 20 July 2022.
